# A Rare Case of Acute Calcium Pyrophosphate Deposition Disease Following Zoledronic Acid Infusion

**DOI:** 10.7759/cureus.44892

**Published:** 2023-09-08

**Authors:** Roxana Florica, Marta B Sekh

**Affiliations:** 1 Internal Medicine, Western Reserve Health Education, Warren, USA; 2 College of Medicine, American University of Antigua, Osbourn, ATG

**Keywords:** cppd, bisphosphonates, pseudogout, zoledronic acid, calcium pyrophosphate deposition disease (cppd)

## Abstract

Calcium pyrophosphate deposition (CPPD) disease of the joints is a form of arthritis that can present in a severely debilitating form of pseudogout. Although mostly idiopathic, pseudogout has been reported following bisphosphonate therapy in only nine cases to date with a pathophysiology that remains unclear. We present the case of a 59-year-old postmenopausal woman who developed the rare onset of acute polyarticular CPPD disease following zoledronic acid infusion for the treatment of osteoporosis.

## Introduction

Calcium pyrophosphate deposition (CPPD) disease is often asymptomatic but may also present as debilitating monoarticular or polyarticular arthritis. Pseudogout is one of the clinical manifestations of CPPD disease. Clinically pseudogout presents with pain, warmth, erythema, and swelling of the affected joint, along with systemic inflammatory signs and symptoms. Radiologically, it presents with chondrocalcinosis of the affected joint. Pseudogout is often idiopathic but can also be due to familial CPPD, trauma, and metabolic and endocrine disorders such as hypophosphatasia, hypomagnesemia, hyperparathyroidism, and hemochromatosis [1,2]. Bisphosphonate (BP) administration is also linked to an increased risk of acute CPPD disease [3].

BPs are widely used to manage osteoporosis, Paget’s disease of the bone, and several malignancies that metastasize to the bone. They inhibit osteoclast-mediated bone resorption and reduce the risk of osteoporotic bone fractures [4,5]. A common side effect associated with BPs is gastrointestinal intolerance following an oral formulation. Intravenous (IV) administration of BPs may cause an acute phase reaction following 24 to 72 hours after an IV infusion characterized by flu-like symptoms and musculoskeletal pain. The more feared side effects include jaw osteonecrosis, atypical femur fracture, atrial fibrillation, and esophageal cancer [6].

Nine cases of CPPD disease secondary to BP therapy have been reported to date, with only three cases due to zoledronic acid infusion [7-14]. This case report describes a rare onset of acute CPPD disease following zoledronic acid infusion for the treatment of osteoporosis.

## Case presentation

A 59-year-old postmenopausal female with a past medical history of osteoporosis, chronic osteoarthritis of the knees, right anterior cruciate ligament repair (twice in 1997 and 2001) after a mechanical fall on right knee, breast cancer (intraductal carcinoma) in 2005 status post-double mastectomy and in remission, gastric ulcer and bypass in 2001, genetic thrombotic disorder (PAI14G5G variant), and asthma presented to the emergency department (ED) with severe right knee pain and difficulty ambulating. The patient received her first zoledronic acid (Reclast) 5 mg IV infusion for the treatment of osteoporosis one day before this visit. Of note, previous oral formulations (Alendronate/Fosamax) were not well tolerated by the patient. She woke up the following morning with severe arthralgia initially affecting only the right knee, with subsequent rapid progression of symptoms to the left knee and right wrist. The patient was seen by her primary care provider that morning and was administered an intra-articular methylprednisolone acetate 40 mg injection to the right knee. Notably, she had responded well to corticosteroid injections for osteoarthritis in the past. Nonetheless, as the right knee pain increased in severity as the day progressed, she was advised to present to the ED for further evaluation.

On evaluation, the patient was hemodynamically stable. She was in severe discomfort, and her right knee was evidently swollen compared to the left, with decreased passive and active flexion and extension secondary to pain. Her right wrist and left knee were mildly swollen, with minimal decreased range of motion. There was no erythema or warmth noted on the physical examination. Laboratory blood tests (Table 1) showed no evidence of leukocytosis with white blood cell (WBC) count at 5600/µL, elevated inflammatory markers such as C-reactive protein (CRP) at 117 mg/L, and erythrocyte sedimentation rate (ESR) at 24 mm/hour, and normal uric acid levels at 4.4 mg/dL. The eosinophil manual count was reported at 0%. Moreover, our patient’s lab work before zoledronic acid infusion showed serum calcium 10.2 mg/dL (reference range: 8.6-10.4 mg/dL) and albumin 5.0 mg/dL (reference range: 3.6-5.1 mg/dL), which dropped to serum calcium of 7.3 mg/dL and albumin of 4.1 mg/dL following the infusion. The patient received several doses of fentanyl 50 µg IV for pain control in the ED which alleviated the pain while at rest; however, the pain was intolerable with palpation and while ambulating.

**Table 1:** Laboratory values on the first day of admission.

Hematology and serum chemistry	Patient’s laboratory value after zoledronic acid infusion	Reference ranges
White blood cell count	5,600 cells/µL	4,500–11,000 cells/µL
Eosinophil count (manual)	0%	n/a
Erythrocyte sedimentation rate	24 mm/hour	0–20 mm/hour
C-reactive protein	117 mg/L	0–4 mg/L
Uric acid	4.4 mg/dL	0–6 mg/dL
Alkaline phosphatase	95 U/L	30–120 U/L
Albumin	4.1 mg/dL	3.4–5.0 mg/dL
Calcium, total serum	7.3 mg/dL	8.2–10.2 mg/dL
Phosphorus	2.4 mg/dL	2.3–4.7 mg/dL
Magnesium	2.3 mg/dL	1.3–2.1 mg/dL
Ferritin	182	15–200 ng/mL

**Figure 1:**
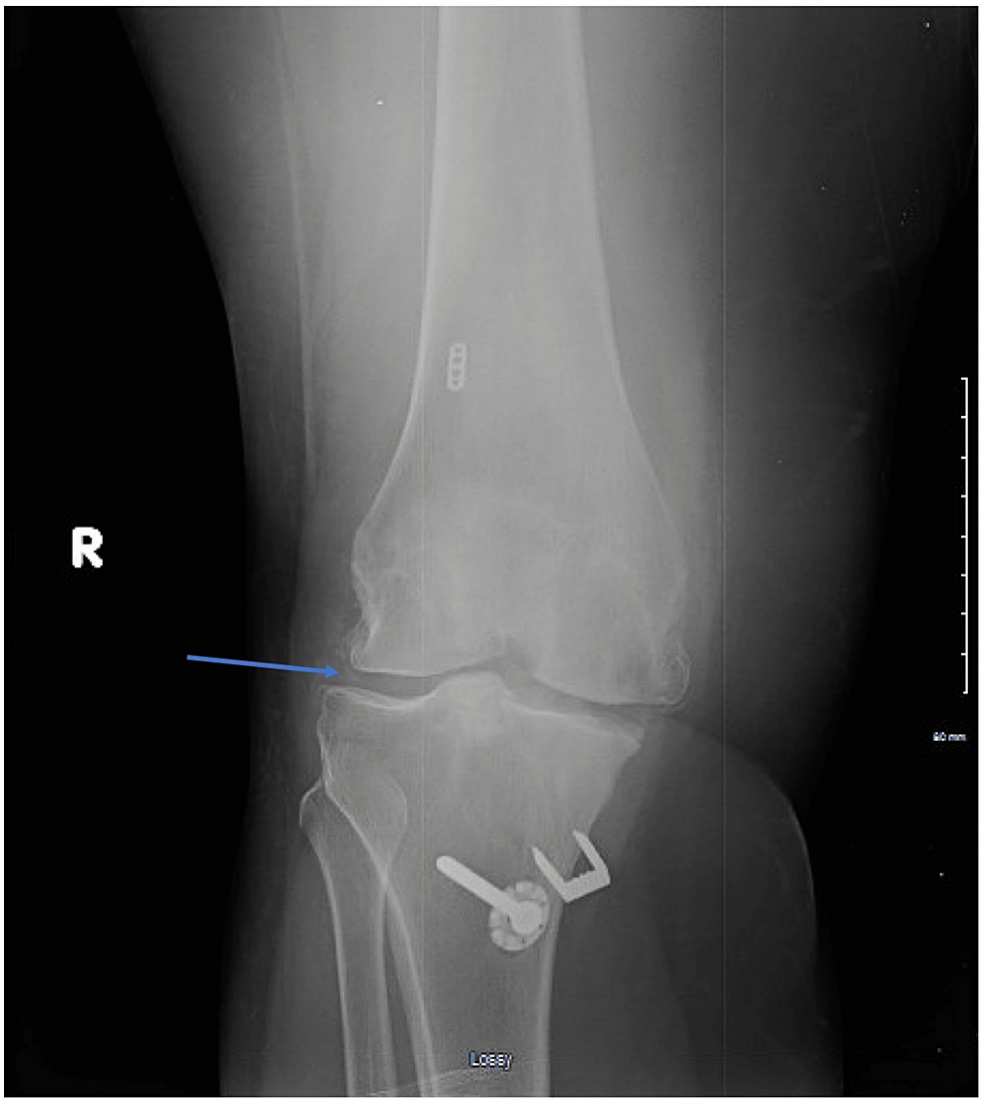
Plain radiograph of the right knee demonstrating mild meniscal chondrocalcinosis due to calcium pyrophosphate dihydrate deposition.

Radiographic findings of the right knee showed advanced arthritis, which was most severe in the medial compartment, and mild chondrocalcinosis (Figure 1). A 70 cc of turbid yellow synovial fluid was aspirated from the right knee joint and rhomboid-shaped calcium pyrophosphate crystals were identified under polarized light optical microscopy. Gram stain of the synovial fluid revealed many WBCs and no organisms, and bacterial fluid cultures were negative. Given the patient’s history of the genetic thrombotic disorder, a venous duplex ultrasound was performed on bilateral lower extremities which identified a deep vein thrombosis (DVT) within the right mild calf veins at the posterior tibial branch. The patient had no previous history of blood clots and was not on any anticoagulation. She was started on apixaban (Eliquis) for DVT management. For the management of acute pseudogout, the patient did not receive non-steroidal anti-inflammatory drugs (NSAIDs) due to her history of gastric ulcers and current DVT diagnosis. She did receive intra-articular injections of 20 mg of triamcinolone acetonide (Kenalog) into each knee and was started on prednisone 40 mg oral tablets for five days with tapering doses (30 mg for five days, followed by 20 mg for five days, followed by 10 mg for five days, followed by 5 mg for five days). At the one-month follow-up, the patient reported complete resolution of swelling and pain and returned to baseline ambulation level.

## Discussion

Our patient presented with CPPD disease within 24 hours following zoledronic acid IV infusion which was confirmed by synovial fluid analysis of the right knee joint that identified CPPD rhomboid-shaped positively birefringent crystals. A plain radiograph of the right knee showed mild chondrocalcinosis and advanced arthritis; however, chondrocalcinosis is difficult to appreciate radiographically in joints with severe cartilage loss. Moreover, signs of systemic inflammation were indicated by elevated CRP and ESR. Before diagnostic CPPD crystal identification, differential diagnoses such as acute gout attack, allergic reaction to zoledronic acid, and septic arthritis were considered. They were ruled out given the normal serum uric acid levels, absence of leukocytosis and eosinophilia, and negative culture and gram stain of the synovial fluid. The physical findings were more indicative of CPPD disease due to the absence of erythema and warmth of the affected joints with severe pain and restricted range of motion.

The literature search identified nine reported cases of CPPD disease secondary to BP therapy. From the reported cases, three were due to zoledronic acid [13,14], two due to pamidronate [8,11], two due to etidronate disodium [7,10], one due to alendronate [9], and one due to neridronic acid [12]. In the reported cases, the age of individuals ranged from 63 to 84 years old, except in two cases that occurred in 47-year-old and 53-year-old individuals. The prevalence of chondrocalcinosis is 3.7% in the population aged 55-59 years and 17.5% in those aged 80-84 years [15]. There is a strong positive association between articular chondrocalcinosis and age which is significant to consider when diagnosing an elderly patient with an acute CPPD disease [16]. Notably, our patient was 59 years old, the age group with a smaller prevalence of chondrocalcinosis, making this a rare event. Another significant factor worthy of attention was the history of meniscectomy reported in the same joint as the CPPD disease following zoledronic acid infusion [13], analogous to our case report. One study showed that chondrocalcinosis is five times more common in knee joints that underwent surgery decades ago than those that were never treated with surgery [17]. BP administration has been shown to increase the risk of acute CPPD disease [3], but it is possible that BPs trigger acute CPPD disease in patients who are already predisposed to it rather than causing the disease itself.

The pathophysiology of CPPD disease secondary to BP administration remains largely unclear, but their temporal relationship is evident; seven out of nine previously reported cases occurred within one week of BP administration. One working theory is that BPs cause a sudden drop in serum calcium, which leads to calcium precipitate in the joints causing acute pseudogout. We found that five out of nine reported cases of CPPD disease following BPs had a drop in serum calcium after the BP administration. This mechanism has been reported to occur following parathyroidectomy in primary hyperparathyroidism, where 25% of patients developed pseudogout postoperatively [18]. As reported previously, our patient lab work showed normal serum calcium and albumin levels before zoledronic acid infusion which significant drop following the infusion. Another possible explanation is that BPs inhibit alkaline phosphatase due to their analogous structure to pyrophosphate, leading to impaired CPPD crystal breakdown resulting in their accumulation in the joints [19,20]. These are only speculatory mechanisms of CPPD disease following BP administration, and more empirical research is required.

## Conclusions

This case report adds to the growing evidence of a rare acute CPPD disease secondary to BP administration. While BPs present a comprehensive treatment option for many skeletal disorders, greater clinical awareness of the risk factors and signs of adverse reactions is required to promote detection and appropriate management. Our case report underscores the importance of continuous surveillance following zoledronic acid infusion for patient safety and optimal therapeutic outcomes even in low-risk populations.
